# A Case of Severe Epstein–Barr Virus-Associated Hemophagocytic Lymphohistiocytosis Successfully Treated With High Dose Steroids and Ganciclovir

**DOI:** 10.7759/cureus.25952

**Published:** 2022-06-15

**Authors:** Pravash Budhathoki, Zaheer A Qureshi, Anish Shah, Sneha Khanal, Haider Ghazanfar, Ahmad Hanif

**Affiliations:** 1 Internal Medicine, Department of Medicine, BronxCare Hospital Center, Icahn School of Medicine at Mt. Sinai, Bronx, USA; 2 Internal Medicine, Icahn School of Medicine at Mount Sinai, New York, USA; 3 Internal Medicine, BronxCare Health System, Bronx, USA

**Keywords:** epstein- barr virus, lymphadenopathy, autoimmune, epstein barr, hemophagocytic lymphohistiocytosis (hlh)

## Abstract

Hemophagocytic lymphohistiocytosis (HLH) is a life-threatening hematological disorder characterized by immune dysregulation with multiple organ involvement and carries a poor prognosis. The occurrence of HLH can be familial or sporadic, which is triggered by causes like infection or malignancy. This case report is about a 47-year-old male who presented to the hospital with a fever, chills, night sweats, and unintentional weight loss. He was found to have severely elevated ferritin, and computed tomography showed cirrhosis, a normal sized spleen, and retroperitoneal lymphadenopathy. He underwent an extensive battery of tests to identify the etiology. Meanwhile, he had recurrent fevers with worsening transaminitis and septic shock, requiring admission to the ICU. Blood tests for Epstein-Barr virus (EBV) deoxyribonucleic acid (DNA) and immunoglobulin G (IgG) were positive. Due to high suspicion of HLH, he was started on intravenous methylprednisone 1000 mg daily for three days with clinical improvement. A bone marrow biopsy showed hemophagocytosis and he was diagnosed with EBV-associated HLH. He was continued on steroids with oral prednisone and continued to clinically improve. He was later tapered off steroids over the course of five months. HLH is a rapidly progressive and fatal condition that requires prompt treatment, and thus a high index of suspicion is needed to make a timely diagnosis.

## Introduction

Hemophagocytic lymphohistiocytosis (HLH) is a life-threatening hematological disorder characterized by immune dysregulation with multiple organ involvement and poor prognosis [1]. The occurrence of HLH may be familial or sporadic, or secondary causes like infection or malignancy can trigger it [2]. It is divided into primary or familial HLH and secondary HLH. Primary or familial HLH is an autosomal recessive disorder, while secondary HLH occurs after a strong immunological disorder, which can be due to immunodeficiency, infection, or underlying malignancy [3]. The most common cause among them is Epstein-Barr virus (EBV) infection [4]. HLH is often challenging to diagnose, and laboratory findings are nonspecific. A high index of suspicion is needed for diagnosis early in the disease course. Here, we present an interesting case of an adult with pyrexia of unknown origin and cytopenia who deteriorated clinically without any definite diagnosis despite extensive workup. It also describes how early intervention based on clinical suspicion altered his clinical course.

## Case presentation

Our patient is a 47-year-old male who presented to the hospital with chief complaints of fever and generalized weakness for two months. The fever was low grade per patient and was associated with chills, night sweats, anorexia, and nausea with occasional episodes of non-bilious non-bloody vomiting. He had an unintentional weight loss of about 30 pounds over the last two months. He also reported non-bloody diarrhea for the same duration. He denied any significant abdominal pain or tenesmus. He also reported having similar symptoms of lesser severity every year around springtime for over 20 years. He had not sought medical attention for his symptoms prior to the current visit and relied on home and herbal remedies to relieve symptoms before. His past medical history was significant for hypertension and diabetes mellitus. He did not have any significant surgical history. Social history included occasional binge alcohol drinking, with the last use being two weeks prior to presentation, but no history of smoking, illicit drug use, or sexually transmitted diseases. The family history was negative for any known malignancy or hematologic disorder.

On initial assessment, the patient was febrile (temperature 38.7 °C) with a blood pressure of 123/75 mmHg, a heart rate of 98 beats per minute, and a respiratory rate of 18 breaths per minute. He was oriented to time, place, and people. He had bilateral vesicular breathing on lung auscultation with no additional sounds. A cardiac examination revealed normal S1 and S2 heart sounds. An abdominal examination revealed a non-tender belly with no evidence of hepatosplenomegaly. The neurological examination was unremarkable.

His initial laboratory workup revealed transaminitis, leukopenia, thrombocytopenia, elevated erythrocyte sedimentation rate (ESR), high C-reactive protein (CRP), and hyperferritinemia with a ferritin level >200,000 ng/ml. This has been presented in Table 1.

**Table 1:** Initial laboratory test results from peripheral blood

Investigation	Value	Reference Range
White blood cell (k/ul)	2.7	4.8–10.8
Lymphocyte count (k/ul)	0.6	1.0–4.8
Neutrophil count (k/ul)	1.8	1.5–8.0
Eosinophil count (k/ul)	0.0	0.05–0.25
Hemoglobin (g/dl)	13.0	12.0–16.0
Hematocrit (%)	36.6	42.0–51.0
Platelet (k/µl)	145	150–440
Sodium (mEq/l)	121	135–145
Potassium (mEq/l)	3.9	3.5–50
Bicarbonate (mEq/l)	17	24–30
Chloride (mEq/l)	86	98–108
Glucose (mg/dl)	130	70–120
Blood urea nitrogen (mg/dl)	22	70–120
Creatinine (mg/dL)	1.3	8–26
Calcium (mg/dL)	8.4	0.5–1.5
Albumin, serum (g/dl)	3.6	3.4–4.8
Total bilirubin (mg/dl)	1.3	0.2–1.2
Conjugated bilirubin (mg/dl)	0.7	0.0–0.3
Alkaline phosphatase (unit/l)	35	53–128
Aspartate transaminase (unit/l)	430	9–48
Alanine aminotransferase (unit/l)	121	5–40
Total protein, serum (g/dl)	6.7	6.0– 8.5
Triglyceride (mg/dL)	393	58–150
Epstein-Barr virus deoxyribonucleic acid quantitative reverse transcription polymerase chain reaction (copies/mL)	71171	<200
Epstein-Barr virus viral capsid antigen immunoglobulin M antibody (U/mL)	164	18–21.99
Cytomegalovirus deoxyribonucleic acid quantitative polymerase chain reaction (log IU/mL)	<2.30	<2.30
Enterovirus ribonucleic acid qualitative reverse transcription polymerase chain reaction (copies/mL)	Not detected	Not detected
West Nile antibody immunoglobulin M, serum	<0.90	<0.90
Antibody assay, HTLV I & II	Non-reactive	Non-reactive
Hepatitis E antibody immunoglobulin M	Not detected	Not detected

**Figure 1:**
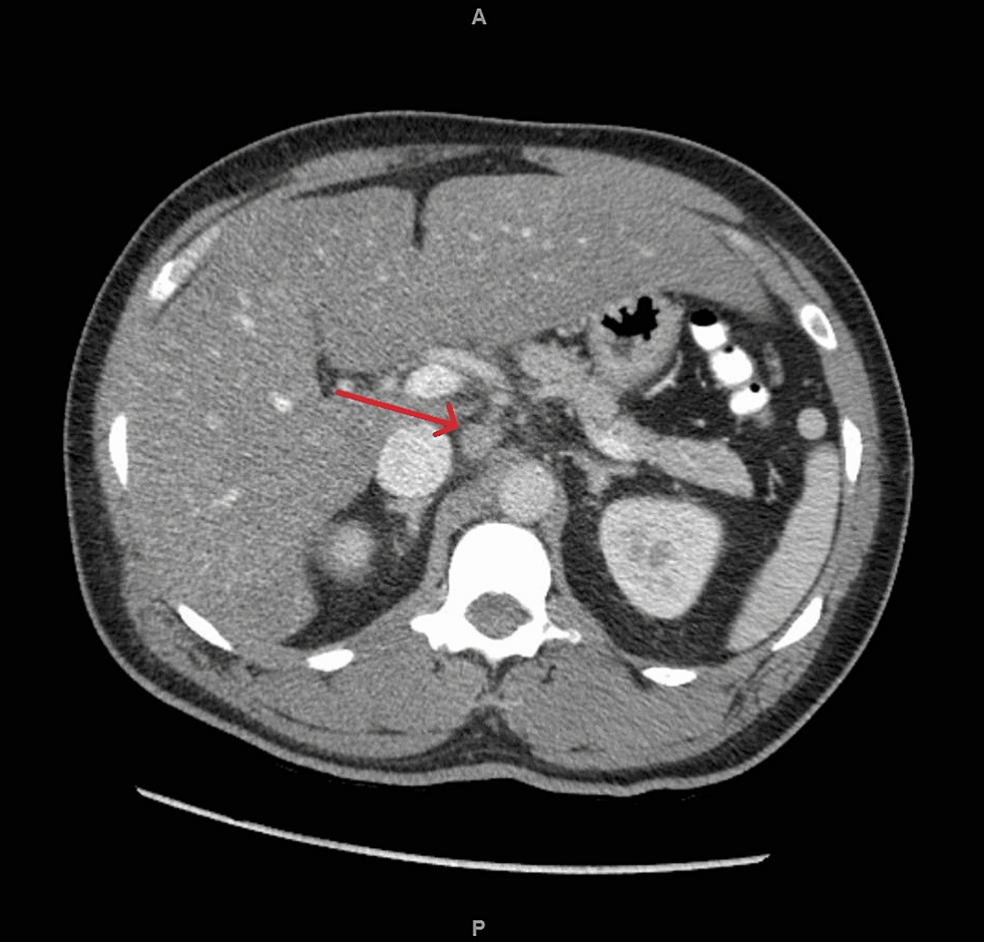
Computed tomography of abdomen and pelvis showing enlarged lymph nodes (red arrow) in upper retroperitoneum and gastric hepatic ligament

An X-ray of the chest was negative for any acute pathology, and an echocardiogram showed an ejection fraction of 59%. As shown in Figure 1, computed tomography (CT) of the abdomen and pelvis showed a fatty liver with possible cirrhosis, a normal spleen, and mild retroperitoneal lymphadenopathy.

The blood culture, urine culture, and serologies for tick-borne diseases and hepatitis panel were negative. The results were negative for antinuclear antibody (ANA), smooth muscle antibody, ceruloplasmin, and immunoglobulin G (IgG) level tests. Peripheral blood smears did not reveal any atypical cells. Ultrasound of the abdomen and magnetic resonance cholangiopancreatography (MRCP) showed a normal liver and pancreas without evidence of obstruction.

The size and location of the retroperitoneal lymph nodes were small and not amenable to biopsy. Due to persistent fevers and transaminitis, the patient was started on empiric treatment with doxycycline for suspected leptospirosis and tick-borne diseases, but no clinical improvement was noted after five days. Kikuchi Fujimoto syndrome was one of the differentials given lymphadenopathy, fevers, night sweats, and abdominal pain. Despite optimal treatment, the patient remained febrile with worsening transaminitis. A bone marrow (BM) biopsy was performed, but the results came back as inconclusive.

On day 8 of admission, he became hypotensive and required management in the intensive care unit (ICU). His labs showed worsening pancytopenia with an absolute neutrophil count (ANC) of 1.1. Epstein-Barr virus viral capsid antigen (EBV-VCA) IgG was found to be elevated (164 U/ml), and EBV DNA came back positive with 71171 copies/ml. These labs indicate EBV reactivation without any known cause. He was started on broad-spectrum antibiotics, including meropenem 1 g IV thrice a day, doxycycline 100 mg IV twice a day, and antivirals including ganciclovir 365 mg IV twice a day. Due to the high clinical suspicion of HLH, he was started on a pulse dose of methylprednisolone 1 g once a day for three days. Thereafter, an improvement in ESR, CRP, and serum ferritin was noted, and the patient became afebrile.

**Figure 2:**
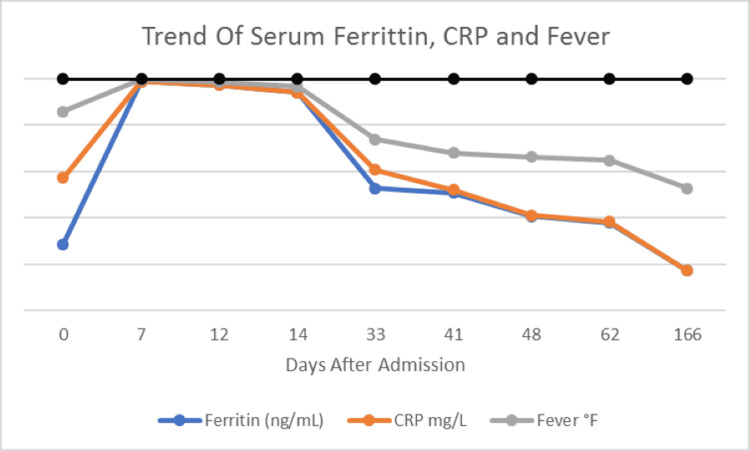
Trend of serum ferritin level, C-reactive protein, and fever (blue arrow indicates the date when steroids were started)

A repeat BM biopsy confirmed the presence of hemophagocytosis, and a formal diagnosis of HLH was made. His ferritin level normalized and, on day 15 of hospitalization, the patient was discharged home on a tapering dose of oral prednisone with the final diagnosis of EBV-associated hemophagocytic lymphohistiocytosis. IL-2 receptor alpha (CD25) soluble level came back as 10,700 pg/mL (normal 532-1892 pg/mL), which was high and favored the diagnosis of HLH. The patient was followed up in the clinic, and prednisone was tapered off slowly over five months with significant improvement in symptoms. The trend of ferritin, CRP, and fever has been presented in Figure 2.

## Discussion

HLH is a rare and aggressive syndrome characterized by excessive inflammation and tissue destruction due to excessive immune activation [5]. The absence of normal downregulation by activated macrophages and lymphocytes is believed to cause hyperinflammatory immune status resulting in activation of macrophages and cytotoxic cells, leading to increased production of inflammatory cytokines [4]. Patients with HLH may present with a single episode or relapses. The usual trigger for an acute episode is an infection or alteration in immune homeostasis like an autoimmune disease. Common infectious agents leading to HLH include EBV, followed by other viruses like cytomegalovirus (CMV), human immunodeficiency virus (HIV), human herpesvirus 8 (HHV8), and parvovirus [6]. Other triggers include inherited syndromes, malignancy, rheumatologic disorders, and HIV infections.

HLH is common in children and young adults, but it may occur at any age. It usually presents as a febrile illness similar to infections, pyrexia of unknown origin, hepatitis, or encephalitis with multiorgan involvement. Common presentations include fever, rash, hepatosplenomegaly, lymphadenopathy, hypotension requiring vasopressors, diarrhea, and neurological symptoms like seizures, ataxia, or altered mental status [7,8]. Kikuchi syndrome is most commonly associated with cervical lymphadenopathy, but rarely, retroperitoneal lymph nodes can also be involved. Patients can either have a prolonged hospital course or clinical deterioration without a clear diagnosis before evaluating for the possibility of HLH. Laboratory abnormalities include cytopenias, high serum ferritin, deranged liver function tests, increased triglycerides, and abnormal coagulation parameters like d-dimer and hemophagocytosis on the bone marrow aspirate [7,9]. The sensitivity of bone marrow in HLH diagnosis is low (60%) [10].

The diagnosis of HLH is based on the criteria used in the HLH-2004 trial, which can be based on genetic mutations or a combination of clinical and laboratory abnormalities. The genetic alterations consistent with a diagnosis of HLH include pathologic mutations of PRF1, UNC13D, Munc18-2, Rab27a, STX11, SH2D1A, or BIRC4. Clinical diagnosis of HLH can be made if five of the following nine findings are present: fever 38.5 °C, splenomegaly, bicytopenias (at least two of the following: hemoglobin C<9 g/d; platelets <100,000/microor absolute neutrophil count <1000 μ/L), hypertriglyceridemia (fasting triglycerides >265 mg/dL) and/or hypofibrinogenemia (fibrinogen <150 mg/dL), hemophagocytosis (in bone marrow, spleen, lymph node, or liver), low or absent NK cell activity, ferritin >500 ng/mL, and elevated soluble CD25 (soluble IL-2 receptor alpha).

Treatment of HLH depends on the clinical status of the patient. Clinically stable patients are usually treated for the triggering factor, while acutely ill patients benefit from HLH-94 protocol treatment using etoposide and dexamethasone at tapering doses over eight weeks [11,12]. HLH associated with EBV is associated with high rates of progression and a worse prognosis [13]. Active EBV infection with more than 10,000 copies of EBV/mcg cellular DNA warrants treatment, and rituximab is sometimes used for this indication. Refractory or recurrent disease is treated with eplamumab and dexamethasone [14]. Our patient has EBV-associated HLH, which was treated with a high-dose steroid plus ganciclovir. Steroids were very effective at controlling the inflammation, with a rapid decline of inflammatory markers, which started worsening when he was briefly off of steroids as an outpatient due to non-compliance. Despite therapy, poor prognosis factors include age, CNS involvement, and remission failure.

Our patient is an adult who presented with pyrexia of unknown origin with weight loss and diarrhea for a prolonged period. The patient had cytopenias, transaminitis, elevated ferritin, lymphadenopathy, and hypotension requiring ICU admission, and the potential trigger was EBV infection, leading to elevated titers of EBV antibody. The patient quickly benefited from steroid treatment and antiviral treatment. Our case highlights the importance of a high index of clinical suspicion with the goal of early initiation of treatment in patients with HLH. Patients with prolonged fever, cytopenia, splenomegaly, and hepatitis with increased ferritin levels resembling a sepsis-like picture without identifying an infectious cause should be kept on a high index of clinical suspicion for HLH. Early intervention becomes especially important given that studies have shown that mortality from HLH can be as high as 58% [15]. At the same time, identification and treatment of triggers are essential, especially in adults with HLH, as HLH in adults is usually secondary and treatment of triggers like autoimmune disease, infection, and malignancy helps prevent a recurrence [10].

## Conclusions

The association between EBV and HLH is common. Our patient’s BM biopsy shows hemophagocytosis. This case emphasizes that HLH should be high in differential even in an unusual presentation. It is possible that the patient could benefit from earlier steroid treatment. Standard management of EBV-associated HLH includes etoposide, high-dose steroids with or without IVIG, and rituximab, but in this patient, only high-dose methylprednisolone along with ganciclovir produced a dramatic clinical response, and thus further therapy was withheld.

Currently, no guidelines for treating HLH in adults are available as the HLH-2004 trial was performed in a pediatric population. Further studies and randomized trials are needed for HLH cases in adults to better understand the pathophysiology, which may help in improving the high mortality of this disease.
